# SPCE-Based Electrochemical Immunosensor for Influenza A (H1) Detection in Serum and Nasopharyngeal Samples

**DOI:** 10.3390/bios16060312

**Published:** 2026-06-01

**Authors:** Mónica D. Garza-Villegas, Itza E. Luna-Cruz, Azael A. Cavazos-Jaramillo, Juan M. Mora-Hernández, Reyes Tamez-Guerra, Cristina Rodríguez-Padilla, Juan M. Alcocer-González

**Affiliations:** 1 Universidad Autónoma de Nuevo León, UANL, Facultad de Ciencias Biológicas, Depto. Inmunología y Virología, Laboratorio de Inmunología, Unidad C, Av. Universidad S/N, Cd. Universitaria, San Nicolas de los Garza 66455, Nuevo León, Mexico; monica.garzavll@uanl.edu.mx (M.D.G.-V.); itza.lunacrz@uanl.edu.mx (I.E.L.-C.); azael.cavazosjrm@uanl.edu.mx (A.A.C.-J.); reyes.tamezgr@uanl.edu.mx (R.T.-G.); cristina.rodriguezpd@uanl.edu.mx (C.R.-P.); 2 SECIHTI, Departamento de Ecomateriales y Energía, Facultad de Ingeniería Civil, Universidad Autónoma de Nuevo León, UANL, Av. Universidad S/N Ciudad Universitaria, San Nicolás de los Garza 66455, Nuevo León, Mexico; jmorah@secihti.mx

**Keywords:** biosensor, immunoelectrochemical, influenza A, SPCE, AuNPs

## Abstract

Acute respiratory diseases caused by viral pathogens such as Influenza A continue to represent a major global health challenge, emphasizing the need for rapid, sensitive, and accessible diagnostic tools. In this work, a carbon screen-printed electrode (SPCE)-based electrochemical immunosensor for the detection of an Influenza A (H1) antigen is reported, incorporating a comparative electrochemical evaluation of four electrode materials. Fe_3_O_4_ nanoparticles, Fe_3_O_4_@C nanoparticles, graphene quantum dots (GQDs), and gold nanoparticles (AuNPs) were systematically assessed by cyclic voltammetry to evaluate their electrocatalytic performance. The highest electrochemical response was selected for biosensor construction. The immunosensor was fabricated by immobilizing antibodies on a modified SPCE and characterized using differential pulse voltammetry (DPV). A concentration-dependent response was observed for H1 antigen concentrations ranging from 0 to 300 ng/mL, with a minimum detectable concentration (MDC) of 1 ng/mL and limit of detection (LOD) of 176 ng/mL and 45 ng/mL for serum and nasopharyngeal swabs, respectively. The biosensor performance was specifically evaluated in complex biological fluids, demonstrating reproducible performance and moderate selectivity against non-target influenza subtypes. Overall, this study highlights the critical role of electrode material selection in determining electrochemical immunosensor performance and supports the potential of SPCE-based platforms for the screening of an Influenza A (H1) antigen in point-of-care-oriented applications.

## 1. Introduction

Some respiratory viruses have been responsible for global outbreaks throughout history, causing significant health and economic losses. Among these viruses, Influenza A is a special concern due to its highly contagious nature and high mortality rate produced by the rapid mutation of surface proteins and the easily airborne infection mechanism [1,2]. The surface glycoprotein, hemagglutinin (HA), has gained special attention because it plays a crucial role in viral infection, mediating the binding to sialic acid receptors on host cells that allows for viral entry. In addition, the H1 subtype of hemagglutinin has been extensively studied not only for its role in viral pathogenicity but also as a key diagnostic marker due to its high mutation rate that allows for easy viral typification [3,4].

Historically influenza viruses have been responsible for several global outbreaks: the first pandemic caused by Influenza A viruses was the “Spanish flu”, caused by the H1N1 subtype and reported in 1918, followed by the “Asian flu” caused by the H2N2 subtype in 1957, the “Hong Kong flu” in 1968 caused by the H3N2 subtype, and the most recent in 2009 caused by the H1N1pdm09 subtype, which resulted in approximately 575,400 deaths worldwide [5,6]. The continuous evolution of the virus through antigenic drift and shift reveals the need for robust diagnostic methods capable of detecting emerging strains [7,8,9].

Among the molecular diagnostic techniques, reverse transcriptase–polymerase chain reaction (RT-PCR) is positioned as the gold standard to detect influenza virus genomic material in a sensitive way [10,11]; however, the expensive costs of the necessary infrastructure for its application, the need for highly qualified personnel and the long response times evidence the need to develop new strategies for the detection and monitoring of viral agents [12,13]. In this way, electrochemical biosensors are positioned as tools with high potential to solve these needs due to their qualities of portability, sensitivity, reproducibility, and specificity [10,14,15]. A biosensor can be defined as an analytical device that, through biochemical reactions mediated by enzymes, immunosystems, organelles, tissues, or cells, can detect chemical compounds through electrical, thermal, or optical signals [16,17,18]. In recent years, viral detection reports based on immunoelectrochemical biosensors have grown significantly, due to the development of new platforms such as screen-printed electrodes. The incorporation of screen-printed electrodes in biosensor mechanisms has made it possible to simplify their design and production processes, improving qualities such as simple surface modification with electrocatalytic nanomaterials and reducing the sample amount needed to perform an analysis [19,20,21]. Despite numerous electrochemical immunosensors reported for influenza detection, direct comparative studies evaluating different nanomaterial-modified SPCEs under identical experimental conditions remain limited. In many cases, reported performance differences arise not only from the intrinsic properties of the nanomaterials but also from variations in electrode geometry, fabrication protocols, electrolyte composition, or signal acquisition parameters. Moreover, many reported systems rely on complex fabrication strategies that compromise reproducibility and scalability. As a result, the rational selection of electrode materials for reproducible and scalable sensor design remains challenging. Therefore, this study aims to construct an immunoelectrochemical device for the screening of the H1 protein of Influenza A H1N1, provide a controlled electrochemical comparison of four nanomaterial-modified SPCEs under identical conditions, and validate the selected platform for immunosensing of Influenza A (H1) in protein-rich biological matrices such as nasopharyngeal swabs and human serum samples.

## 2. Materials and Methods

### 2.1. Reagents and Materials

Iron (II) sulfate heptahydrate (FeSO_4_·7H_2_O), iron (III) hexahydrate (FeCl_3_·6H_2_O), chloroauric acid (HAuCl_4_), sodium citrate, citric acid, bovine albumin serum, 1-ethyl-3-(3-dimetilaminopropil) carbodiimide (EDC), *N*-hydroxysuccinimide (NHS), and potassium ferrocyanide (K_4_Fe(CN)_6_) were purchased from Sigma Aldrich (St. Louis, MO, USA). PBS was purchased from Thermo Fisher Scientific (Waltham, MA, USA). H1 and H5 proteins, and antibodies were purchased from SinoBiological (Beijing, China). Screen-printed carbon electrodes (SPCEs) were purchased from Metrohm DropSens (Oviedo, Spain).

### 2.2. Equipment

Electrochemical detections were performed using a 910 PSTAT mini (Metrohm, Herisaw, Switzerland), and SEM analyses were performed using a JEM 6390-LV JEOL microscope (JEOL Ltd., Tokyo, Japan).

### 2.3. Fe_3_O_4_ Nanoparticle, Fe_3_O_4_@C Nanoparticle, Graphene Quantum Dot (GQD), and Gold Nanoparticle (AuNP) Synthesis

Fe_3_O_4_ nanoparticles were synthesized via the coprecipitation method described by Bae et al. (2012) [22]. Briefly, 202.6 mg of iron (II) sulfate heptahydrate (FeSO_4_·7H_2_O) and 546.1 mg of iron (III) chloride hexahydrate (FeCl_3_·6H_2_O) were dissolved in 50 mL of ddH_2_O. Then, 15 mL of ammonium hydroxide was added to obtain a black nanoparticle solution, which was incubated at 60 °C for 2 h under magnetic stirring. The synthesis was carried out under an argon atmosphere. The resulting nanoparticles were washed sequentially with ddH_2_O, ethanol, and acetone using an external magnetic field, followed by drying at 25 °C for 12 h. For Fe_3_O_4_@C nanoparticles, the carbon shell was obtained through a hydrothermal process at 180 °C for 12 h using 300 mg of curcumin as the carbon source.

GQD synthesis was performed according to the method reported by Zhu et al. (2021) [23]. Briefly, 2 g of citric acid was heated at 180 °C for 120 min. The obtained solution was added dropwise to 30 mL of NaOH (10 mg/mL) under magnetic stirring. The GQD solution was stored at 4 °C until use.

The synthesis of AuNPs was carried out through a typical chemical reduction method. Here, 20 mL of 1 mM HAuCl_4_ was heated at 100 °C for 10 min under magnetic stirring, followed by the addition of 1 mL of 1% sodium citrate under continued stirring. The mixture was then incubated for an additional 10 min.

### 2.4. Nanomaterial Characterization

All synthesized nanomaterials were characterized by transmission electron microscopy (TEM) using the JEOL JEM 2011 model microscope and images of scanning electron microscopy (SEM) were obtained using the JEOL JEM 6390-LV model microscope (JEOL Ltd., Tokyo, Japan).

### 2.5. Electrochemical Characterization of Nanomaterials

The electrocatalytic properties were evaluated through an SPCE. The working electrode of 4 mm was coated with 10 µL of the immobilized nanomaterials via drop-casting or electrodeposition. The SPCE was incubated for 24 h at 25 °C. Electrochemical measurements were performed by recording cyclic voltammetry (CV) scans within a potential window of −0.4 to 0.9 V at a scan rate of 50 mVs^−1^. A 1X PBS solution (pH 7.4, Thermo Fisher Scientific) was used as the electrolyte, and measurements were conducted using a potentiostat (910 PSTAT mini Metrohm), performed in triplicate (*n* = 3). The optimal electrocatalytic material was selected for biosensor construction.

### 2.6. Biosensor Construction and Characterization

**Figure 1:**
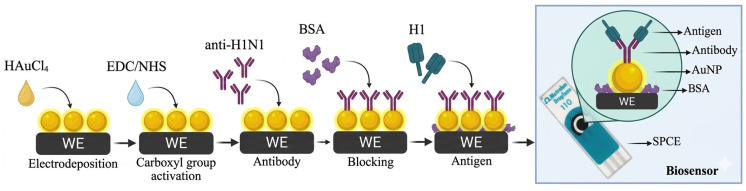
Diagram of biosensor construction.

Influenza A H1N1 antibody (SinoBiological) was used to obtain nanoparticle–antibody nanocomplexes using modified Wahyuni et al. (2018) [24] and Chin et al. (2017) methods [25]. For nanocomplex formation, 30 µL of 100 mM 1-ethyl-3-(3-dimethylaminopropyl) carbodiimide (EDC) and 50 mM N-hydroxysuccinimide (NHS; Sigma-Aldrich) were added to activate the nanoparticles’ carboxyl groups to enable covalent immobilization of antibodies via amide bond formation with the amino groups of the biomolecules [26,27]. The mixture was incubated for 24 h at 25 °C to facilitate conjugation and to ensure efficient formation of reactive NHS esters promoting stable covalent attachment. Subsequently, 5 μL of antibody (100 μg/mL) was added to the activated nanoparticles to promote WE coverage and ensure effective antigen–antibody interactions. To prevent nonspecific binding, 2 μL of 1% bovine serum albumin (BSA; Sigma-Aldrich) was added as a blocking agent [28]. In Figure 1, we can observe the diagram that represents the biosensor construction procedure proposed in this work.

### 2.7. Immunoelectrochemical Detection of H1 Protein

Each development stage of the biosensor was electrochemically evaluated through CV measurements, in a potential range between −0.4 and 0.6 V, at a scanning rate of 50 mVs^−1^. For final biosensor evaluation, the DPV technique was employed in a potential range of −0.6 to 0.6 V, at a scanning rate of 50 mV/s^−1^, using potassium ferrocyanide (K_4_Fe(CN)_6_, 10 mM and KCl 100 mM) as the electrolyte. To evaluate the sensor performance across a diverse set of independent samples to better reflect real-world variability, five samples of serum and ten of nasopharyngeal swabs were assessed once by incubating 10 µL of clinical samples with known concentrations of hemagglutinin (H1) (0, 1, 50, 100, 200, and 300 ng/mL) for 20 min at 25 °C prior to measurement. Finally, the minimum detectable concentration (MDC) was defined as the lowest antigen concentration experimentally distinguishable from the blank signal under the tested conditions, and the limit of detection (LOD) was calculated for both biological samples using the standard 3σ/slope method.

### 2.8. Biosensor Specificity Test

Once the correct development of the biosensor and the optimal detection of Influenza A H1N1 hemagglutinin/HA protein on human samples were established, a specificity test was performed using H5 protein at the previously mentioned concentrations, and the obtained currents were compared to previous recorded currents from HA protein detections.

## 3. Results

### 3.1. Nanomaterial Morphological Characterization

**Figure 2:**
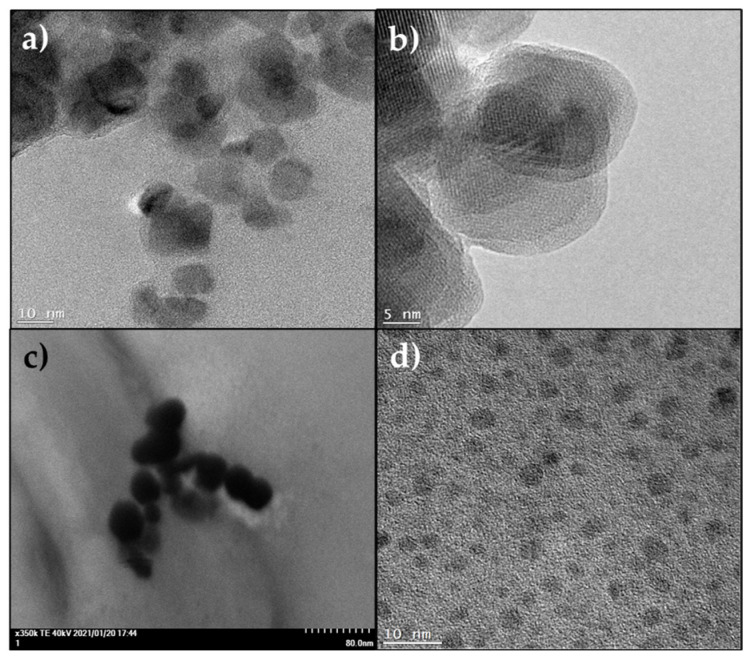
Transmission electron microscopy (TEM). Synthesized (**a**) Fe_3_O_4_; (**b**) Fe_3_O_4_@C; (**c**) Au; (**d**) GQD nanoparticles. Scale bar = 10 nm.

The synthesized nanomaterials (Fe_3_O_4_ and Fe_3_O_4_@C, AuNPs, and GQDs) were characterized by transmission electron microscopy (TEM), scanning electron microscopy (SEM), and energy-dispersive X-ray spectroscopy (EDS). Figure 2a shows that the Fe_3_O_4_ magnetic cores exhibited a cubic-rectangular crystal structure with an average length of 10 nm. In contrast, Figure 2b illustrates Fe_3_O_4_ magnetic cores coated with carbon derived from curcumin with a nearly spherical morphology and an average diameter of 15 nm. Figure 2c displays gold nanoparticles (AuNPs) with sizes ranging from 35 to 40 nm and a near-spherical shape. Finally, the graphene quantum dots (GQDs) were analyzed by TEM, revealing well-dispersed, uniform spherical dots with diameters between 4 and 5 nm (Figure 2d).

### 3.2. Synthesized Nanomaterials Electrochemical Characterization

**Figure 3:**
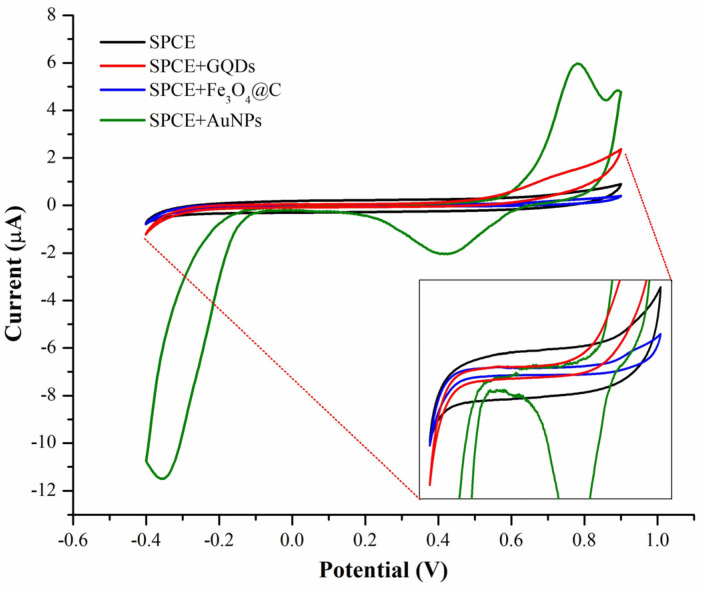
Cyclic voltammogram analysis of the electrocatalytic properties of the SPCE and nanomaterials. Potential between −0.4 and 0.9 V, at a scanning rate of 50 mVs^−1^, using 1X PBS, pH 7.4, as the electrolyte.

The Fe_3_O_4_@C nanoparticles, AuNPs, and GQDs immobilized over the working electrode (WE) of the SPCE were evaluated using CV to assess their electrocatalytic activity for potential application as an electrocatalytic material on an Influenza A H1N1 virus biosensor (Figure 3). The lowest faradaic currents (0.39 µA) exhibited by the bare SPCE and SPCE + Fe_3_O_4_@C are indicative of limited electroactive surface area and inefficient charge transfer. In contrast, immobilized GQDs demonstrated a higher anodic current (2.36 µA), reflecting improved electron transfer kinetics and increased availability of electroactive sites. Notably, AuNPs exhibited the most promising results, showing the highest faradaic current in both cathodic and anodic regions (5.97 µA), or 2.5-fold higher compared to that of GQDs and under identical conditions, making them suitable for facilitating electron transfer processes relevant for biosensor construction. The Fe_3_O_4_ nanoparticles did not show a difference compared to the bare SPCE. Based on these findings, we selected the SPCE-AuNP matrix for subsequent biosensor development.

### 3.3. Modified SPCE Characterization

**Figure 4:**
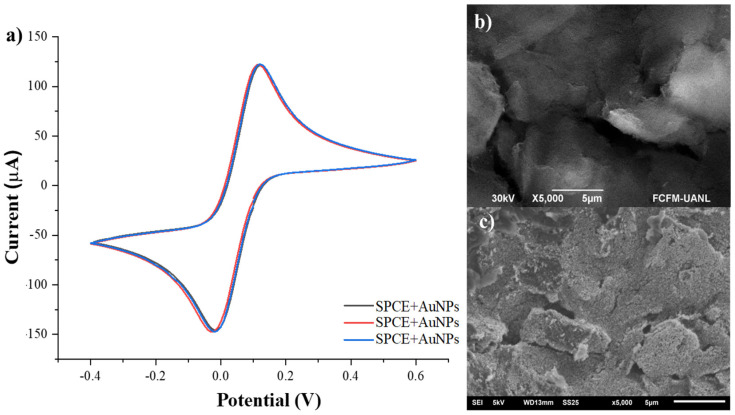
(**a**) Cyclic voltammogram of SPCE modified with AuNPs. Potential between −0.4 and 0.9 V, at scanning rate of 50 mVs^−1^, using potassium ferrocyanide (K_4_Fe(CN)_6_, 10 mM and KCl 100 mM) as electrolyte. Scanning electron microscopy (SEM); (**b**) Bare SPCE; (**c**) SPCE modified with AuNPs. Scale bar = 5 µm.

To assess the repeatability of SPCE modification with AuNPs, three SPCEs were electrodeposited with AuNPs, and their electrocatalytic properties were analyzed using CV. The modified SPCEs showed peak currents of 121.48, 121.82, and 122.61 µA, respectively, with nonspecific signals detected (Figure 4a). Furthermore, SEM images of the unmodified SPCE and the AuNP-modified SPCE revealed a uniform distribution of nanoparticles across the electrode surface (Figure 4b,c).

**Figure 5:**
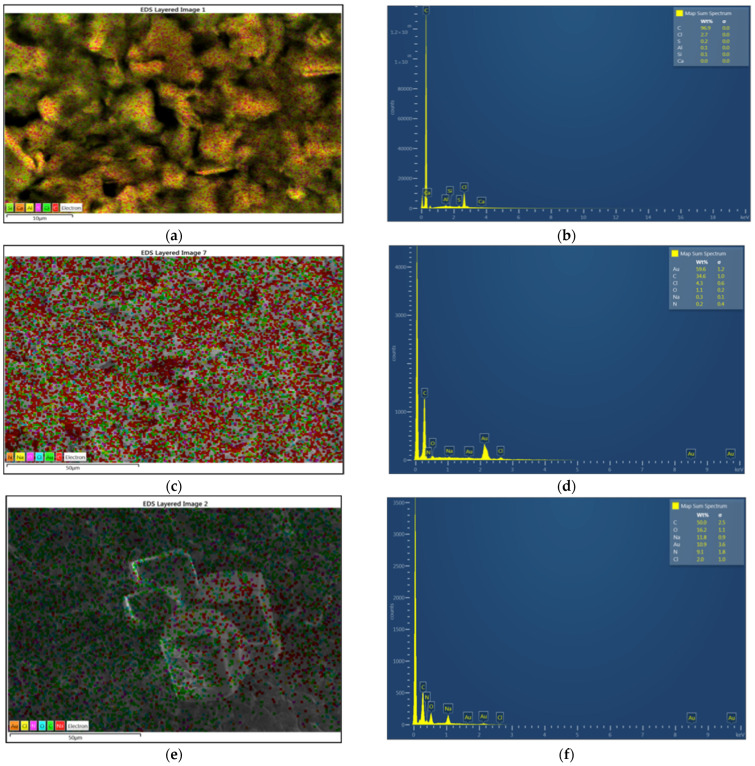
Scanning electron microscopy (SEM) and EDS of (**a**) bare SPCE; (**b**) chemical composition of SPCE; (**c**) SPCE modified with AuNPs; (**d**) chemical composition of SPCE modified with AuNPs; (**e**) SPCE modified with AuNPs and antibody anti-H1N1; (**f**) chemical composition of SPCE modified with AuNPs and antibody anti-H1N1.

To confirm the conjugation of immobilized AuNPs with the H1N1 antibody on the SPCE, an SEM-EDS analysis was carried out. Figure 5a shows the bare SPCE, where SEM images reveal a surface composed of carbon layers. The corresponding EDS analysis in Figure 5b identifies carbon and oxygen as the predominant elements on the electrode surface. In contrast, Figure 5c shows the working electrode (WE) of SPCE modified with AuNPs, with the chemical composition analysis in Figure 5d indicating 59.6% gold, highlighting the successful modification compared to the unmodified SPCE. In Figure 5e, the SEM analysis of the modified working H1N1 antibody–AuNP–SPCE shows no discernible differences in AuNP morphology. However, the EDS spectrum in Figure 5f confirms the presence of the antibody, evidenced by 9.1% nitrogen originating from the H1 protein monoclonal antibody.

**Figure 6:**
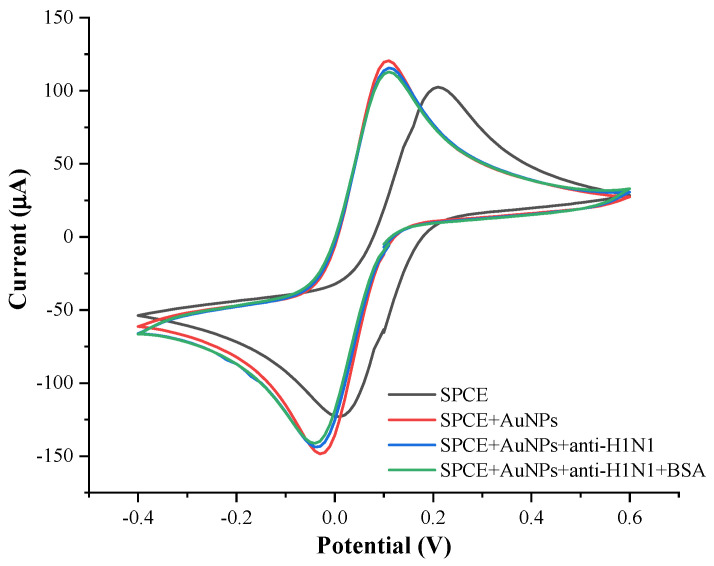
Cyclic voltammogram of biosensor characterization to detect H1. Potential between –0.4 and 0.6 V, at scanning rate of 50 mVs^−1^, using potassium ferrocyanide (K_4_Fe(CN)_6_, 10 mM and KCl 100 mM) as electrolyte.

To assess the correct SPCE modification and corroborate an adequate electrochemical response, a CV test for the Influenza A H1N1 virus was carried out for the bare SPCE, SPCE + AuNPs, SPCE + NCs, and SPCE + NCs + BSA. The maximum oxidation peak for the bare SPCE reached a current of 102.44 µA; meanwhile, the SPCE + AuNPs reached a value of 120.60 µA. It is crucial to understand that the addition of the antibody for H1N1 and the blocking agent promotes a decrease in the oxidation current peak, such that this effect is the signal that corroborates the expected interaction of the biosensor (Figure 6).

### 3.4. Immunoelectrochemical Detection of Influenza A H1N1 Virus Hemagglutinin

**Figure 7:**
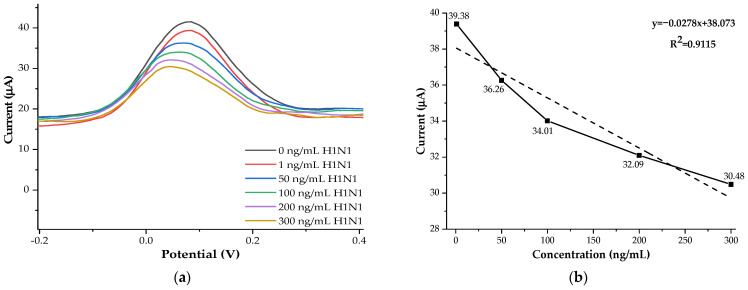
(**a**) Differential pulse voltammetry of hemagglutinin detection for Influenza A H1N1 virus. Potential between –0.4 and 0.6 V, at scanning rate of 50 mVs^−1^, using potassium ferrocyanide (K_4_Fe(CN)_6_, 10 mM and KCl 100 mM) as electrolyte; (**b**) Linear regression analysis.

An immunoelectrochemical detection assay of the hemagglutinin protein of the Influenza A virus was performed. Figure 7a shows the DPV analysis for PBS supplemented with hemagglutinin concentrations. The measurements reveal an oxidation peak with a maximum current value of 41.5 µA for the H1N1 subtype. Notably, the current values decrease as the protein concentration increases, with maximum oxidation peak values recorded at 39.38 µA, 36.26 µA, 34.01 µA, 32.09 µA, and 30.48 µA for H1N1 at concentrations ranging from 0 to 300 ng/mL, yielding a correlation coefficient (R) of 0.9115 (Figure 7b).

### 3.5. Immunoelectrochemical Detection of Influenza A H1 Protein on Biological Samples

**Figure 8:**
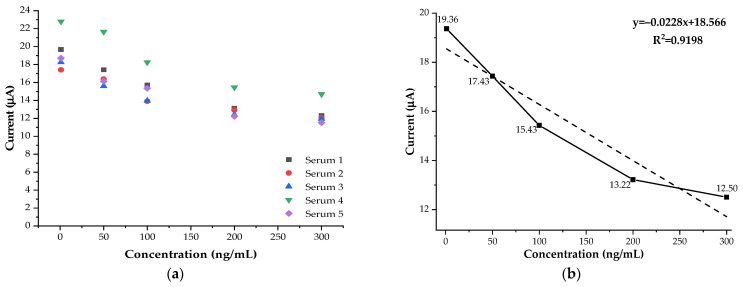
(**a**) IP graph of 5 serum samples supplemented with H1 protein from the patients analyzed using the H1 biosensor; (**b**) Linear regression analysis.

**Figure 9:**
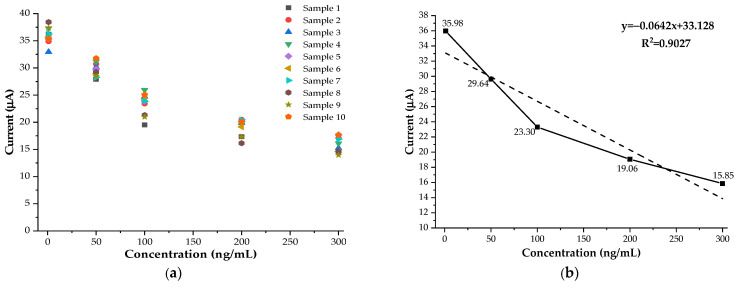
(**a**) IP graph of 10 nasopharyngeal swab samples supplemented with H1 protein from the patients analyzed using the H1 biosensor; (**b**) Linear regression analysis.

In Figure 8 and Figure 9, we observe the high repeatability and sensitivity of our biosensor. The current values for the maximum oxidation peak were highly consistent in serum and nasopharyngeal swab samples. Figure 8 demonstrates that the biosensor could detect H1 protein concentrations in serum within a range of 1 to 300 ng/mL, with oxidation peak values ranging from 19.36 to 12.5 µA. In contrast, Figure 9 shows that nasopharyngeal swabs exhibited a more stable electrochemical response under the evaluated conditions. DPV analysis of these samples showed less variation in current values for the same H1 concentrations, in contrast to serum samples. The oxidation peaks for these samples ranged from 35.98 to 15.85 µA.

Based on these analyses, we determined that the MDC of the biosensor was 1 ng/mL, and the LOD was estimated at 176 ng/mL and 45 ng/mL for serum and nasopharyngeal swabs, respectively.

In Table 1 we analyze the performance of the immunosensor in biological samples through statistical analysis. The results are presented as mean ± standard deviation, along with 95% confidence intervals. For serum samples, the measured electrochemical signal decreased progressively with increasing hemagglutinin concentration, from 21.02 ± 1.64 µA at 0 ng/mL to 12.50 ± 1.24 µA at 300 ng/mL. A similar trend was observed for nasopharyngeal swab samples, where the signal decreased from 51.75 ± 1.47 µA at 0 ng/mL to 15.86 ± 1.39 µA at 300 ng/mL. Recovery values in serum ranged from 62.5% to 96.8%, while in swab samples they ranged from 39.6% to 90.0%. The calculated 95% confidence intervals remained relatively narrow across all concentrations, indicating good reproducibility of the measurements in both biological matrices.

### 3.6. Immunoelectrochemical Biosensor Specificity Assay

**Figure 10:**
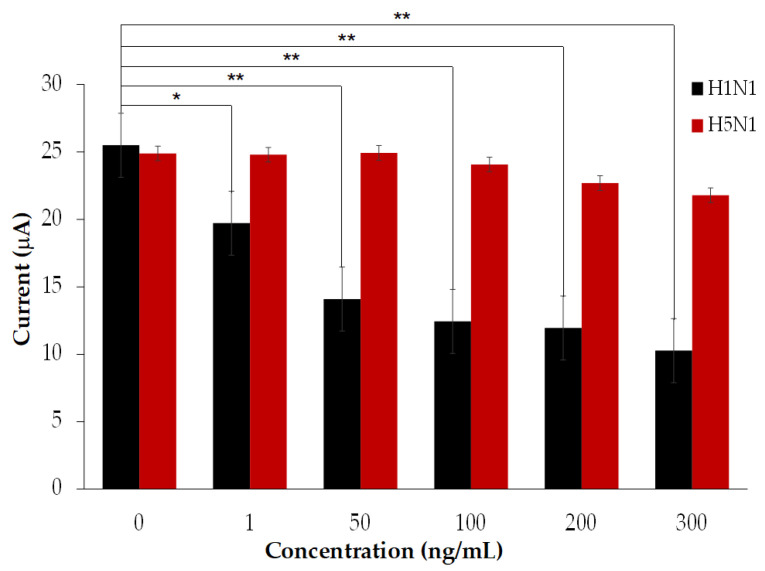
Biosensor specificity analysis in human serum samples supplemented with hemagglutinin H1 and H5. One-way ANOVA (* *p* < 0.005, ** *p* < 0.001), Tukey’s post hoc test.

With the biosensor specificity assay, we demonstrate that as the concentration of the target protein increases, the anodic current (I) decreases significantly, ranging from 25.49 to 10.27 µA. In contrast, when analyzing the HA protein of the H5N1 subtype, the current shows only a slight non-significant decrease, from 24.88 to 21.79 µA (Figure 10). The response toward H1N1 is approximately five-fold higher than that observed for the non-target H5 protein, indicating moderate selectivity.

## 4. Discussion

The analytical performance of the proposed electrochemical immunosensor was systematically evaluated through electrochemical characterization, calibration studies, and selectivity assays in both controlled and complex biological matrices. The observed decrease in anodic current upon antigen binding is consistent with the formation of an immunocomplex on the electrode surface, which partially hinders electron transfer of the redox probe. This response mechanism has been previously reported in electrochemical immunosensors and supports the reliability of the signal transduction strategy employed in this work.

Figure 3 shows the cyclic voltammograms obtained using a screen-printed carbon electrode (SPCE) and after its modification with graphene quantum dots (SPCE + GQDs), magnetite nanoparticles (SPCE + Fe_3_O_4_@C), and gold nanoparticles (SPCE + AuNPs). In the negative potential region (−0.4 to 0.0 V), all systems exhibit predominantly capacitive currents. However, the bare SPCE (black curve) shows a more stable response with lower current density, whereas the modified electrodes display a slight increase in background current, indicating an enhancement of the double-layer capacitance [29]. The GQD-modified electrode exhibits a moderate increase in current compared to the bare SPCE, while maintaining a similar voltametric profile. This suggests that its primary contribution arises from an increased electroactive surface area rather than a strong electrocatalytic effect. Similarly, the Fe_3_O_4_@C-modified electrode shows a comparable response, although with a slight decrease in current within certain potential regions, which may be associated with lower intrinsic conductivity or a less efficient distribution of active sites on the electrode surface [30]. In contrast, the AuNP-modified electrode exhibits markedly different behavior. A significant increase in both anodic and cathodic currents is observed, along with the appearance of a well-defined cathodic feature around ~0.4 V. This suggests the presence of additional faradaic processes or a greater contribution of electroactive sites facilitated by the gold nanoparticles. Furthermore, in the positive potential region (>0.6 V), the AuNP-modified electrode exhibits a sharp increase in anodic current, indicating significantly enhanced charge-transfer kinetics [31]. This behavior contrasts with the more gradual current increase observed for GQDs and Fe_3_O_4_@C, as well as the limited response of the bare SPCE. Overall, the detailed analysis of both the capacitive region and the extreme potential ranges indicates that incorporating nanomaterials increases the electrochemically active surface area [32], while AuNPs notably improve conductivity and promote more efficient electrochemical processes.

Several conjugation methods have been reported, but EDC-NHS coupling remains one of the most used techniques for biosensor construction due to its simplicity in linking antibodies to nanoparticles. In this study, we employed these agents for conjugation to couple the antibody to the gold nanoparticle surface. In addition to this kind of conjugation, the intrinsic properties of AuNPs facilitate electrostatic interactions between their negatively charged surface and the positively charged antibodies, as illustrated in Figure 5 [33]. The successful antibody immobilization was confirmed through chemical composition analysis. Even after repeated electrode washing cycles, the presence of nitrogen (a characteristic element exclusively derived from the antibodies) was detected, verifying their stable binding to the nanoparticle surface. The decrease in current observed in cyclic voltammetry can be directly attributed to the progressive surface blocking of the screen-printed carbon electrode (SPCE) during biosensor fabrication. After modification with nanomaterials, the electrode surface exhibits enhanced electrochemical activity due to increased electroactive area and improved electron transfer. However, subsequent functionalization steps significantly alter this behavior. Specifically, the immobilization of antibodies via EDC/NHS chemistry, followed by blocking with bovine serum albumin (BSA), forms an insulating organic layer on the electrode surface. These biomolecules are inherently non-conducting and partially cover the electrode’s active sites, hindering access of the redox probe to the surface. As a result, electron transfer between the electrolyte species and the electrode becomes less efficient, leading to a decrease in faradaic current. Additionally, the physical barrier created by the biomolecular layer increases charge-transfer resistance and reduces the effective electrochemically active surface area. Overall, the observed reduction in current serves as an electrochemical signature of successful surface functionalization and biomolecule immobilization, confirming the stepwise construction of the biosensor [34,35,36,37].

In this context, the lowest antigen concentration experimentally distinguishable from the blank was defined as the MDC, which was determined to be 1 ng/mL. This experimentally defined metric was selected as a realistic metric that provides a practical assessment of sensor performance, particularly in complex biological matrices or protein-rich matrices, rather than emphasizing a theoretical limit of detection.

A continuous decline in oxidation peak values was observed as the hemagglutinin protein concentration increased (0–300 ng/mL), consistent with previously reported immunoelectrochemical detection systems. This phenomenon can be explained by the accumulation of antigen–antibody complexes on the SPCE surface, which hinders electron transfer between the electrode and the electrolyte. As highlighted in earlier analyses, the antibody used in this study lacks electroactive sites (Figure 7) [26,38,39].

In the analysis of supplemented serum samples (Figure 8), a concentration-dependent decrease in the electrochemical signal was observed for serum H1 protein concentrations between 0 and 300 ng/mL, consistent with an inhibition-based detection mechanism. A negative result was determined when the current exceeded 20 µA in the absence of H1 protein. Similarly, Figure 9 showed a comparable concentration-dependent behavior for nasopharyngeal swabs, with negative results indicated by oxidation currents above 50 µA. These results, confirmed with the analysis statistics observed in Table 1, demonstrate that the proposed immunosensor provides a consistent and concentration-dependent signal for both biological samples and they reflect the nonlinear relationship between signal inhibition and antigen concentration, commonly reported in electrochemical immunosensors as stochastic biomolecular interactions. The difference in oxidation currents between serum and swab samples can be attributed to matrix effects. The higher concentration of endogenous serum proteins, such as albumin, has been identified as a primary non-electroactive component that attenuates the current in immunoelectrochemical biosensors. In contrast, the lower protein content in nasopharyngeal swab samples likely contributes to improved electron transfer and enhanced sensitivity. Despite the presence of matrix effects, the narrow confidence intervals observed across all concentrations indicate a stable and reproducible biosensor [40,41].

Finally, the selectivity of the immunosensor was evaluated using H1N1 as the target antigen and H1N5 as the non-target protein. While H1N1 produced a clear concentration-dependent decrease in the electrochemical signal, H1N5 induced only minimal signal variations across the same concentration range.

The response variation for H1N1 was approximately five-fold higher than that observed for H5N1, indicating preferential recognition of the target antigen, and confirms that the electrochemical response is predominantly governed by specific antigen–antibody interactions [42].

In Table 2, we compare our biosensors against previously reported influenza electrochemical immunosensors. The reported LOD values vary widely from fg/mL to µg/mL in the order of the biosensing platform’s complexity. Although the LODs obtained with our systems are not the lowest reported for ultrasensitive systems, they are representative of SPCE-based platforms and are adequate for practical detection in real biological samples. Moreover, our biosensors demonstrate competitive performance with previously reported work that reached an LOD of 3.54 µg/mL on synthetic saliva [26]. Several reported systems shown in Table 2 were able to obtain lower LODs in comparison to our system; nevertheless, they rely on more complex systems, such as microfluidic systems, specific nanomaterial surface immobilization, or multiple functionalization stages that increase fabrication time, cost, and operational complexity. In contrast, our SPCE-based platform maintains competitive performance with a simpler design. We recognize that future optimization to lower our LODs is required; nevertheless, successful detection of H1 protein nasopharyngeal swabs and saliva matrices has rarely been evaluated at the same time in previous works, and it represents an important step toward real clinical applicability.

## 5. Conclusions

The present study describes the development of an electrochemical immunosensor for the detection of Influenza A (H1) hemagglutinin based on screen-printed carbon electrodes and a systematic comparison of different nanomaterial electrode modifications. Through electrochemical evaluation under identical experimental conditions, gold nanoparticle-modified SPCEs were identified as the most suitable platform, offering improved electrocatalytic behavior and reproducibility compared to other evaluated materials.

The resulting immunosensor exhibited a concentration-dependent electrochemical response toward the target antigen, with a minimum detectable concentration of 1 ng/mL and stable performance in protein-rich, biological matrices such as human serum and nasopharyngeal swabs. These matrices contain high concentrations of protein, salt, and endogenous electroactive species that can interfere with electrochemical measurements and compromise sensor reliability. Despite these challenges, the developed platform maintained consistent signal response and reproducibility. Although the analytical sensitivity is lower than that reported for some more complex systems, the proposed platform prioritizes simplicity, reproducibility, and compatibility with disposable electrode formats, which are key factors for decentralized and point-of-care screening applications.

## Figures and Tables

**Table 1:** Analytical performance of the immunosensor in biological samples.

Matrix	Spike (ng/mL)	Signal (µA, Mean ± SD)	Recovery (%)	95% CI
Serum	0	21.02 ± 1.64	-	19.2–22.8
Serum	1	19.36 ± 2.04	96.8	16.8–21.9
Serum	50	17.43 ± 2.38	87.2	14.8–20.0
Serum	100	15.43 ± 1.70	77.1	13.4–17.5
Serum	200	13.22 ± 1.36	66.1	11.7–14.7
Serum	300	12.50 ± 1.24	62.5	11.1–13.9
Nasopharyngeal swab	0	51.75 ± 1.47	-	50.6–52.9
Nasopharyngeal swab	1	35.98 ± 1.68	90.0	34.7–37.2
Nasopharyngeal swab	50	29.64 ± 1.25	74.1	28.7–30.6
Nasopharyngeal swab	100	23.70 ± 2.02	59.2	22.2–25.2
Nasopharyngeal swab	200	19.26 ± 1.38	48.1	18.2–20.3
Nasopharyngeal swab	300	15.86 ± 1.39	39.6	14.8–16.9

**Table 2:** Comparison of reported electrochemical immunosensors for influenza detection and the proposed sensor.

Platform	Nanomaterial/Strategy	Target (Analyte)	Clinical Matrix	LOD	Ref.
Gold Screen-Printed Electrode (AuSPE)	MWCNT–Au–Pt + anti-H1	Influenza H1N1 virus	Synthetic saliva	3.54 µg/mL	[26]
Glassy carbon electrode (GCE)	Protein A + anti-His IgG + recombinant His-tagged H5	Anti-H5	Hen serum	2.1 pg/mL	[27]
SPCE	Gold nanoflowers	Hemagglutinin H1	Synthetic saliva	19 pg/mL	[43]
Microfluidic electrochemical chip	Reduced graphene oxide (RGO) + antibody	Influenza H1N1 virus	Clinical respiratory samples (swab-derived)	0.5 PFU/mL	[44]
Gold electrode	AuNPs + antibody	Hemagglutinin H7	PBS buffer	5 µg/mL	[45]
SPCE	AuNPs + anti-H1 + EDC/NHS	Hemagglutinin H1	Serum and nasopharyngeal swab	Serum: 176 ng/mL Nasopharyngeal swab: 45 ng/mL	This work

## Data Availability

The original contributions presented in this study are included in the article. Further inquiries can be directed to the corresponding author.

## Abbreviations

The following abbreviations are used in this manuscript:

AuNPs	Gold nanoparticles
GQDs	Graphene quantum dots
SPCE	Screen-printed carbon electrode
CV	Cyclic voltammetry
DPV	Differential pulse voltammetry
HA	Hemagglutinin
LOD	Limit of detection
TEM	Transmission electron microscopy
SEM	Scanning electron microscopy
EDS	Energy-dispersive X-ray spectroscopy
MDC	Minimum detectable concentration
NCs	Nanocomplexes
